# Multiple Pyoderma Gangrenosum Overlying AV Fistula Treated With Colchicine: A Case Report

**DOI:** 10.1177/20543581241284749

**Published:** 2024-10-05

**Authors:** Alex Derstenfeld, Rosalie-Sélène Meunier, Josée Bouchard, Alexandra Mereniuk

**Affiliations:** 1 Département de Dermatologie, Université de Montréal, QC, Canada; 2 Département de Médecine, Hôpital du Sacré-Coeur de Montréal, QC, Canada

**Keywords:** pyoderma gangrenosum, arteriovenous fistula, chronic kidney disease, dialysis, colchicine

## Abstract

**Rationale:**

Pyoderma gangrenosum (PG) is a rare neutrophilic dermatosis which gives rise to painful ulcers. Pyoderma gangrenosum can be triggered by trauma, a phenomenon called pathergy. Here, we report the first case of PG arising from pathergy due to needle insertion overlying an arteriovenous fistula (AVF). This case report seeks to inform nephrologists about PG, this yet unreported presentation, and management in the context of hemodialysis.

**Presenting concerns:**

A 69-year-old woman presented to dermatology clinic for erythemato-violaceous plaques with central ulceration at the site of needle insertion overlying her AVF. The patient was known for chronic renal insufficiency secondary to C3 glomerulonephritis, for which she received hemodialysis. After an accidental burn which lead to appearance of a painful ulcer, following each needle insertion for hemodialysis, she would develop an erythematous papule that progressed to a painful ulcer with erythematous-violaceous borders.

**Diagnosis:**

Pyoderma gangrenosum was clinically diagnosed and both clinical and paraclinical evaluation did not reveal any secondary cause of PG.

**Intervention:**

Dialysis via AVF was suspended due to the risk of triggering more PG and was temporarily pursued by central venous catheter. The patient was initially treated with prednisone and topical corticosteroids. Furthermore, owing to the high recurrence rate of PG, colchicine was initiated in prevention to avoid resorting to immunosuppressive or long-term corticotherapy.

**Outcomes:**

The patient’s lesions improved on prednisone, which was then tapered over 1 month. Following prednisone taper and continuing improvement of PG on colchicine and topical corticosteroids alone, the decision was taken to recommence dialysis via AVF after performing a negative pathergy test. Topical corticosteroids were ceased due to the risk of cutaneous atrophy and were replaced by pimecrolimus ointment. The patient has continued dialysis via AVF ever since, without recurrence.

**Novel Finding:**

This is the first case reported of PG arising from pathergy due to needle insertion overlying an AVF. Colchicine may be a safe and effective therapy for long-term treatment of PG in the context of hemodialysis.

## Résumé

**Contexte:**

Le Pyoderma gangrenosum (PG) est une dermatose neutrophilique rare qui provoque des ulcères douloureux. Le PG peut être déclenché par un traumatisme, un phénomène appelé pathergie. Nous rapportons ici le premier cas de PG résultant d’une pathergie au site d’insertion de l’aiguille recouvrant une fistule artérioveineuse. Ce rapport de cas vise à informer les néphrologues sur le PG, sur cette présentation non encore rapportée et sur sa prise en charge dans le contexte de l’hémodialyse.

**Présentation du cas:**

Une femme âgée de 69 ans s’étant présentée à une clinique de dermatologie pour soigner des plaques érythémateuses violacées avec ulcération centrale au site d’insertion de l’aiguille recouvrant sa fistule artérioveineuse (FAV). La patiente était connue pour une insuffisance rénale chronique secondaire à une glomérulonéphrite à C3, pour laquelle elle recevait des traitements d’hémodialyse. À la suite d’une brûlure accidentelle ayant entraîné l’apparition d’un ulcère douloureux, la patiente développait une papule érythémateuse qui évoluait vers un ulcère douloureux aux bords érythémateux violacés après chaque insertion d’aiguille pour l’hémodialyse.

**Diagnostic:**

Le Pyoderma gangrenosum a été diagnostiqué cliniquement et l’évaluation, tant clinique que paraclinique, n’a révélé aucune cause secondaire de PG.

**Intervention:**

En raison du risque accru de déclenchement de PG, la dialyse par la FAV a été suspendue et remplacée temporairement par la dialyse par cathéter veineux central. La patiente a d’abord reçu un traitement de prednisone et de corticostéroïdes topiques. En raison du taux élevé de récidive de PG, un traitement préventif à la colchicine a été initié pour éviter le recours à une corticothérapie immunosuppressive ou à long terme.

**Résultats:**

Les lésions de la patiente se sont améliorées avec le traitement à la prednisone, celui-ci a ensuite été réduit progressivement sur une période d’un mois. Après la réduction progressive de la prednisone et en raison de l’amélioration continue du PG avec le traitement par colchicine et corticostéroïdes topiques seulement, la décision a été prise de recommencer la dialyse par la FAV après un test de pathergie négatif. Un risque d’atrophie cutanée a justifié l’arrêt des corticostéroïdes topiques qui ont été remplacés par une pommade de pimecrolimus. Depuis, la patiente a poursuivi sa dialyse par FAV, sans récidive.

**Principales observations:**

Il s’agit du premier cas signalé de PG résultant d’une pathergie au site d’insertion de l’aiguille recouvrant une fistule artérioveineuse. La colchicine peut s’avérer un traitement sûr et efficace pour le traitement à long terme du PG dans le contexte de l’hémodialyse.

## Introduction

Pyoderma gangrenosum (PG) is a rare neutrophilic dermatosis characterized by rapidly progressive painful ulceration with violaceous undermined edges. The pathogenesis of PG remains unknown. However, it is currently believed that they arise due to immune dysfunction, most likely innate in origin. Pyoderma gangrenosum may be idiopathic (50%) or associated with underlying conditions including inflammatory bowel disease (Crohn’s or ulcerative colitis, 20%-30%), arthritis (seronegative arthritis, spondylitis of inflammatory bowel disease, or rheumatoid arthritis, 20%), hematological malignancies/dyscrasias (15%-25%), as well as autoinflammatory syndromes.^1^ New research also demonstrates an increased risk of PG in patients with chronic renal failure and on dialysis.^2^

Initially, PG may present as acneiform pustules or erythematous plaques which typically progress to painful ulcers with undermined edges and erythematous-violaceous borders. Most often, these lesions present on the lower limbs although they may present anywhere on the body. Although classic ulcerative PG is the most common form, there also exist superficial, bullous, pustular, and vegetative variants. Trauma may precipitate lesions in 20% to 30% of cases and is called the pathergy phenomenon.^2^ The pathergy phenomenon remains poorly understood. Lesions may occur at sites of surgery, but pathergy may be triggered by trauma as trivial as an injection site or intravenous access site.

## Presenting Concern

A 69-year-old woman developed C3 membranoproliferative glomerulonephritis in 2016. Remission was obtained with prednisone and mycophenolate mofetil. Over the summer of 2020, the patient’s renal function deteriorated in the absence of apparent trigger. Hemodialysis was first undertaken via right subclavian central venous catheter (CVC). A radio-cephalic arteriovenous fistula (AVF) of the right arm was created in September 2020. Healing of the wound was uneventful. Hemodialysis through AVF was initiated in May 2021. Thereafter, the patient received bi-weekly hemodialysis at her request.

Medical history was notable for an IgG monoclonal gammopathy of undetermined significance (MGUS). The patient’s medications included furosemide, levothyroxine, darbepoetin, famotidine, pregabalin, and vitamin D.

**Figure 1.**
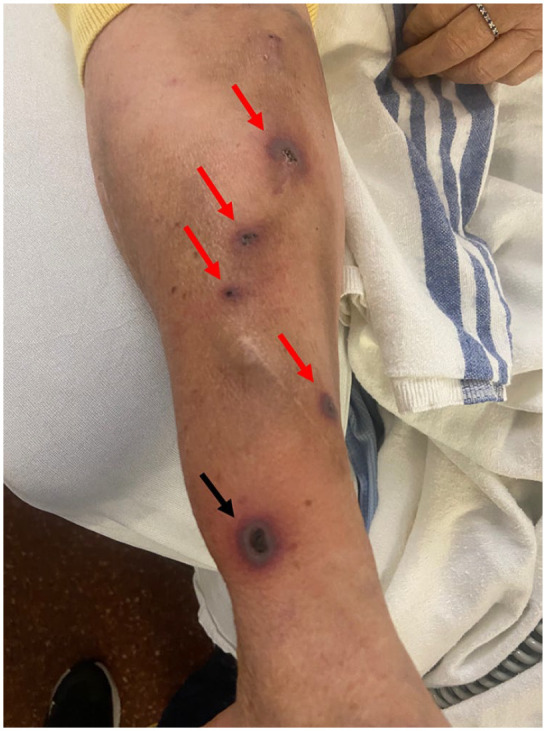
Pyoderma gangrenosum overlying AV fistula. Black arrow: pyoderma gangrenosum overlying initial burn. Red arrows: pyoderma gangrenosum at needling sites overlying AVF.

Following a superficial burn to her right wrist in August 2021, the patient developed bullae which, over the ensuing week, progressed to an ulcer surrounded by erythemato-violaceous borders. Following this, within 48 hours at each hemodialysis needle insertion site the patient developed painful erythematous papulopustules which progressed to ulcers with the same characteristic features (see Figure 1). The lesions raised concern for the ongoing viability of the AVF and the patient was sent for consultation at the combined internal medicine-dermatology clinic.

## Clinical Findings

On clinical examination, a total of 5 lesions were present on dorsal aspect of the patient’s right arm. On the right wrist at the site of the initial burn was a punched-out ulcer with surrounding undermined erythematous-violaceous borders and surrounding erythema. The other 4 lesions had the same morphology arising at the sites of needle insertion into the AVF. Further examination of the patient’s skin demonstrated no other suspect lesions. There was no associated synovitis.

Extensive review of systems revealed no gastrointestinal symptoms, ocular symptoms, or arthralgias. No symptoms of hematological malignancy were present. Moreover, the patient had no fever, no at-risk exposition for atypical infections including no recent travel history, and no risk factors for atypical mycobacterial infections or deep mycoses.

Extensive repeat paraclinical testing showed no evidence of vasculitis and repeat serum protein electrophoresis and serum-free light chains were normal.

## Diagnostic Focus and Assessment

The aspect, distribution, and natural history of the lesions in our patient pointed to PG as the cause of her lesions. The differential diagnosis of ulcerative PG is broad and includes various infections, vascular disease, medium vessel vasculitis including polyarteritis nodosa, granulomatous vasculitis, Beçhet’s disease, as well as cutaneous malignancies.^3^ However, with the exception of certain atypical mycobacterial infections and leishmania, none of the other entities in the differential diagnosis present as multiple ulcers limited to one extremity in a linear distribution. Leishmania was excluded as the patient had not traveled recently. Discrimination between infectious causes and PG was made on the basis that all lesions followed minor trauma. Pyoderma gangrenosum is known to occur after minor local trauma, a phenomenon called pathergy. Here, the fact that the lesions occurred immediately after and at the precise locations of a burn and needle insertion into the AVF were highly suggestive that the lesions arose due to pathergy phenomenon. In addition, the evolution of each lesion from a papulopustule to a painful ulcer with erythemato-violaceous was highly suggestive of PG.

Owing to the high level of confidence in the clinical diagnosis and the risk of triggering a new lesion further compromising the AVF, a cutaneous biopsy for histopathology and tissue cultures was not undertaken. Should the lesions have failed to respond to prednisone, we would have remained open to the possibility of an infectious etiology and would have undertaken a cutaneous biopsy after discussion with vascular surgery.

Moreover, no secondary cause of PG was identified. As the patient did not present with gastrointestinal symptoms, we did not pursue fecal calprotectin or consult gastroenterology for a colonoscopy. The patient had already been assessed for rheumatoid arthritis a year earlier, with negative anti-cyclic citrullinated peptides and rheumatoid factor, and did not present with arthritis or arthralgias. Although 5% of patients with PG are known to have a MGUS, they are most often associated with IgA.^3^ The patient’s IgG MGUS had been stable for years prior to PG and demonstrated normalization with repeat serum protein electrophoresis making its imputation as a cause unlikely.

## Therapeutic Focus and Assessment

**Figure 2.**
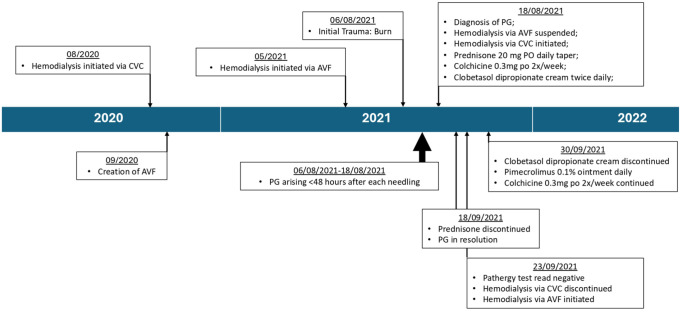
Timeline of clinical events.

Following our consult and diagnosis of PG in August 2021, dialysis via AVF was suspended due to the risk of triggering other PG (see Figure 2). Dialysis was temporarily pursued by CVC. To ensure a rapid response, the patient was initially treated with prednisone 20 mg daily as well as topical class 1 corticosteroids twice daily. As a prompt return to the use of her AVF was required, the decision was made to initiate long-term steroid-sparing therapy to minimize the risk of PG recurrence; colchicine 0.3 mg twice weekly was initiated in hopes to avoiding immunosuppressive therapy. The patient’s lesions improved on prednisone, which was then tapered by 5 mg a week over 1 month. Following prednisone taper, the PG continued to heal on colchicine and topical corticosteroids alone. However, following the taper, the patient began to experience ipsilateral upper limb edema. Ultrasound was preformed and excluded superficial or deep vein thromboses as a cause. The edema was believed to be a complication of dialysis via CVC. As the patient was quickly becoming intolerant to dialysis via CVC, it became necessary to determine whether the patient’s PG were adequately controlled and whether possible to resume dialysis via AVF.

To determine whether the patient was able to resume dialysis via AVF, a pathergy test was conducted. A pathergy test is undertaken by inserting a 20- to 22-gauge needle intradermally at a 45-degree angle, creating minor trauma, and assessing whether a papulopustule forms 48 hours later at the site of needle insertion.^4^ The presence of a papulopustle constitutes a positive test, indicating that the underlying pathology is still active. In September 2021, a pathergy test was undertaken and read as negative. As her condition appeared to be adequately controlled, dialysis via AVF was recommenced the same day.

## Follow-up and Outcomes

Since dialysis at the AVF site was recommenced, the patient has not developed a new PG. Owing to adequate control and lack of new lesions, class 1 topical corticosteroids were changed for pimecrolimus 0.1% ointment daily as maintenance treatment to avoid steroid atrophy. The patient has continued colchicine since that time and has not developed a recurrence of PG.

## Discussion

Cases of PG following postoperative AVF creation or peritoneal catheter insertion have been reported.^5-7^ However, we believe this to be the first reported case of needling-induced PG in a hemodialysis patient. The incidence of PG is increased in chronic kidney disease and among patients on hemodialysis with no causative relationship yet established.

Pyoderma gangrenosum remains a clinical diagnosis. Criteria to establish the diagnosis of PG have been proposed, requiring the presence of 2 major and 2 minor criteria (Table 1), but remains a diagnosis of exclusion.^8^ Other causes of cutaneous ulceration that should be considered include arterial and venous disease, conditions causing vaso-occlusion, infections, vasculitis, calciphylaxis, cutaneous cancers, metastasis, and other inflammatory conditions.

**Table 1.** Proposed Diagnostic Criteria.

**Major criteria**
Rapid progression of a painful necrolytic ulcer with irregular violaceous undermined borders
Exclusion of other causes of cutaneous ulceration
**Minor criteria**
History of pathergy or cribriform scarring
Associated systemic disease (inflammatory bowel disease, arthritis, IgA gammopathy, or underlying malignancy)
Classic histopathological findings
Treatment response (rapid response to systemic steroid treatment)

Presence of 2 major and 2 minor criteria for diagnosis of pyoderma gangrenosum.

A thorough history with a special focus on any underlying inflammatory condition or malignancy is indicated. A complete skin examination should be performed, and a biopsy for histopathology and culture should be considered.

Paraclinical tests should include a complete blood count and C-reactive protein as well as renal function tests, protein electrophoresis, and serum-free light chains. Vasculitis screen, cryoglobulins, fecal calprotectin, and endoscopy should be considered on the basis of clinical suspicion. Routine pre-immunosuppression screening should be undertaken given that PG often requires systemic glucocorticoids and additional immunosuppressive therapy to adequately control the condition.

In the case at hand, the diagnosis was facilitated by both classic clinical appearance of the lesions and the systematic development of a new ulcer after each new trauma. Rapid improvement with prednisone also supported the diagnosis.

There is no official established treatment algorithm for PG. Milder forms can be managed with potent topical corticosteroids while additional intralesional corticosteroid injections can be useful where there are a small number of lesions. Treatment choice is guided by the severity of the disease and patients’ comorbidities. Adjunctive systemic treatment in mild disease may include colchicine, dapsone, or tetracycline antibiotics for their anti-inflammatory effects. Immunosuppressive therapy such as azathioprine, methotrexate, cyclosporine, or mycophenolate mofetil can be considered in the off-label treatment of recalcitrant PG.^9^ Moreover, emerging evidence supports the use of various biologic treatments for PG, including anti-TNF agents, anakinra, ustekinumab, as well as others.^10^ However, there is not yet consensus as to which therapy may be most effective nor when they are indicated.^11^

As our patient had end-stage renal disease, dapsone was not considered due to the risk of hemolysis in the context of pre-existing anemia. Intralesional corticosteroids were excluded owing to the risk of inducing atrophy that could compromise the viability of the AVF. Methotrexate was contraindicated. Should we have had to escalate therapy, mycophenolate mofetil would have been considered which had been well tolerated by the patient during initial treatment of her glomerulonephritis.

Pyoderma gangrenosum overlying AVF constitute a rare complication which may threaten arteriovenous dialysis access. As this exceptional case demonstrates, interruption of local trauma by temporarily suspending the use of AVF and rapid treatment of PG were essential. Our experience has shown that colchicine may be a safe and effective adjunctive therapy for treatment and prevention of PG recurrence in the context of dialysis, and prompt recognition of pathergy and PG was crucial to the overall favorable clinical outcome. No recurrence of PG has been reported 3 years later.
